# Research on the Initiative Scientific Research and Innovation Behavior of Postgraduates: Based on the Theory of Planned Behavior

**DOI:** 10.3389/fpsyg.2022.839981

**Published:** 2022-04-27

**Authors:** Hong Wang, Chunhuai Yang, Meichen Meng, Yaxue Zeng

**Affiliations:** ^1^Tourism School, Hainan University, Haikou, China; ^2^Zhongyuan Design Group, Haikou, China; ^3^Tourism School, Hainan University, Haikou, China

**Keywords:** initiative scientific research and innovation behavior, postgraduates, the theory of planned behavior, scientific research innovation intention, the promotion of achievements in scientific research

## Abstract

To clarify the generative mechanism and influencing factors of graduate students’ willingness and behavior of initiative scientific research and innovation, this research builds a quantitative model consisting of six variables: academic interest, regulatory pressure, conditions for improving scientific research and innovation capabilities, willingness to take initiative in scientific research and innovation, the promotion of achievements in scientific research, and initiative scientific research and innovation behavior. In total, 684 valid questionnaires were distributed and collected through WeChat Moments. Descriptive statistical analysis, reliability and validity analysis, factor analysis, path analysis, and conditioning analysis were conducted using the SPSS and structural equation model (SEM). The results showed that: (1) academic interest, regulatory pressure, and conditions for improving scientific research and innovation capabilities have a positive impact on the intention of initiative scientific research and innovation, but the impact of regulatory pressure is extremely insignificant. (2) The willingness to take initiative in scientific research and innovation has a positive and significant impact on initiative scientific research and innovation behavior, and the promotion of achievements in scientific research plays a negative moderating effect. Finally, this manuscript puts forward the suggestions on how to promote the initiative scientific research and innovation behavior of postgraduate students based on the research results.

## Introduction

Postgraduate education plays an important role in cultivating innovative talents, improving innovation capabilities, serving economic and social development, and cultivating innovative talents required for high-quality economic and social development, emphasized by the Chinese President Xi (2020). In an increasingly competitive society, it is essential to cultivate the creativity of graduate students (Baglin et al., 2017). The scientific research ability is a key indicator to measure the quality of graduate education, and the scientific research and innovation capabilities of postgraduate students is the core component of the innovation ability of graduate students. Building a perfect training system for graduate students’ scientific research and innovation capabilities has increasingly become a major focus of the academic circles (Haddad et al., 2021).

With the profound changes in the China’s economic development mode, the implementation of an innovation-driven national strategy requires the training of a large number of innovative talents. The Ministry of Education of China (MOE) divides higher education levels into: postgraduate (doctorate/master), undergraduate in regular Higher Education Institutions (HEIs), undergraduate in adult HEIs, and undergraduate in online HEIs; among them, master postgraduate students can be divided into master program under central ministries (MOE/other departments), local authorities (MOE/other departments), non-governmental sectors, and Chinese-foreign cooperatively run schools with corporate capacity. There are a total of 2,673,049 postgraduate students of various types. With the expansion of the scale of enrollment, the phenomenon of postgraduate students with academic level of undergraduates has begun to emerge from the water (Wen, 2020). Postgraduates have the problems of low scientific research abilities, poor practical ability, and insufficient innovation ability (Iqbal and Mahmood, 2010).

Postgraduates’ own factors are an important constraint on the cultivation of their scientific research capabilities, such as learning methods, cognitive ability, and volitional quality (Tao et al., 2020). At the same time, tutors and their training design are undoubtedly the key factors influencing the cultivation of postgraduates’ scientific research capabilities. Factors, such as social capital of graduate students’ classmates and family members (Brodin, 2018), the academic community among the students with the same or similar academic pursuits and value orientation, the resulting academic interactions (Meng et al., 2017), the outstanding research ability of the research team leader (Boni et al., 2009), the e-learning adoption (Schneckenberg, 2009), all affect the cultivation of graduate students’ scientific research capabilities. Related research mainly based on the field of pedagogy reveals the law of the cultivation of graduate students’ scientific research capabilities. However, as a microcosmic subject, graduate students’ innovation capacities are dynamic, complex, and diverse. It is very necessary to initiate a discussion based on psychology, behavioral sciences, and other micro-levels, but it has not attracted sufficient attention from scholars.

This manuscript focuses on the microcosmic subjects, innovatively introduces the concept of initiative scientific research and innovation, selects the theory of planned behavior (TPB) and system innovation theory, and complements the moderating effect of the variable “promotion of research achievements” on the basis of TPB model, so as to clarify the generative mechanism and influencing factors of graduate students’ willingness and behavior of initiative scientific research and innovation, and to provide suggestions for effectively stimulating the practice of graduate students’ initiative scientific research and innovation behavior based on the conclusion of quantitative model research.

## Theoretical Background and Hypothesis Development

### Theoretical Background

#### Innovation Theory

Innovation can be divided into technological innovation and institutional innovation. The principle of innovation theory plays a fundamental role in the interpretation of innovative subjects, behaviors, and activities. According to collaborative innovation theory, under the framework of the national innovation strategy system, the four-in-one organizational structure of government, industry, university, and research, the government actively coordinates the organizational resources and integrates innovative subjects with completely different functions, such as universities, enterprises, research institutes, and intermediaries, so as to give full play to their respective advantages and organically integrate into an effective innovative subsystem (Aydin, 2017). Promoting the cooperation of innovative subsystems through the promotion of achievements in scientific research is a powerful means of system innovation in scientific research and innovation.

#### Theory of Planned Behavior

Theory of planned behavior is derived from the theory of reasoned action (TRA), which was proposed by Fishbein and Ajzen to analyze how people change their behavior patterns through personal willingness. Ajzen proposed the TPB. TPB is one of the most persuasive theories for studying behavioral willingness and its antecedents. The theory points out that behavior is the result of its own rational thinking, and its variables include attitude, subjective norms, and perceived behavioral control (Fishbein and Ajzen, 1975). Attitude refers to an individual’s positive or negative feelings toward a behavior, and is the view and evaluation of the implementation of a specific behavior; subjective norms are the views and opinions on the social pressure when engaging in specific behaviors; and perceived behavioral control refers to the degree of ease or difficulty an individual perceives to achieve a particular behavior, which reflects the subject’s perception of factors helping or hindering the behavior and their influence (Rana et al., 2019).

### Hypothesis Development

#### Academic Interest and Willingness of Initiative Scientific Research Innovation

##### Initiative Scientific Research Innovation

Innovation, as inventiveness grounded in field-specific knowledge and expedited by motivation, is related to creativity, novelty, implementation, and entrepreneurship (Tierney and Lanford, 2016). Scholars argued that in his empirical research: the innovation behavior of scientific and technological personnel can be divided into passive and initiative behaviors; spontaneity, preliminary preparation, and overcoming obstacles are the core elements of initiative innovation behavior (Casati and Genet, 2014). Since initiative scientific research and innovation subjects are spontaneous, well-prepared and not afraid of setbacks, their behavior is of great significance to high-quality and valuable scientific research and innovation output.

##### Academic Interest

Academic interest, broadly defined as personal orientations toward activities that are intended to develop one’s academic skills and knowledge (Lee and Durksen, 2018), has been linked to better academic performance (Lee and Shute, 2010). Burch’s student involvement theory states that the effective participation of doctoral students in the scientific research process requires not only their participation at the action level, but also the input of the thinking, which mainly refers to the interest in the process of participating in academic activities; moreover, interest can promote students’ personal development while participating in the scientific research (Burch et al., 2015). Additionally, scholars paid attention and proved that interest is essential to engagement (Bazeley, 2010) and autonomy (Tierney and Lanford, 2016) that are necessary for innovation.

To sum up, as an internal and lasting motivation, academic interest has a positive impact on the willingness to take initiative in scientific research and innovation. For graduate students, the higher the academic interest, the stronger the willingness to take initiative in scientific research and innovation. Based on this, we put forward the following research hypotheses:

Hypothesis 1. Academic interest has a positive and significant impact on the willingness of initiative scientific research innovation.

#### Regulatory Pressure and Willingness of Initiative Scientific Research Innovation

Regulatory elements are important institutional variables, which is a clearly stipulated rule that must be followed. Lots of research showed that institutional variables are closely related to the research productivity (Teodorescu, 2000). The regulatory rules are accompanied by corresponding rewards and punishments. Organizations or members of society respond to them out of instrumental logic rights (Wamala and Ssembatya, 2015). The core manifestation of regulatory elements is policies and rules, which infer the behaviors that “have to do,” and the emotional support for the regulated objects can be fear, guilt, or innocence and ease (Hedjazi and Behravan, 2011; Fang and Fu, 2014). Regulatory pressure refers to the pressure brought on by external policies and regulations that require postgraduate students to carry out scientific research and innovation.

Scholars argue that school management is a key element of the research performance. Management and the culture related to university research work have an impact on the effect of scientific research (Edgar and Geare, 2013). It is stated that the quantity and quality of academic papers published by training units are linked with academic degrees, and the establishment of minimum academic standards for graduation and training plans creates a regulatory environment for scientific research, in which postgraduate students “have to” conform to the regulatory environment and have the motivation to complete their learning tasks of scientific research. Through quantitative research, the author points out that the regulatory pressure has a significant positive impact on graduate students’ research innovation motivation (Li et al., 2021).

To sum up, as an external and mandatory factor influencing graduate students’ willingness to scientific research and innovation, regulatory pressure has a positive impact on their willingness to scientific research and innovation. The greater the regulatory pressure, the stronger the willingness of scientific research and innovation. Based on this, we put forward the following research hypotheses:

Hypothesis 2. Regulatory pressure has a positive and significant impact on the willingness of postgraduates in scientific research and innovation.

#### Conditions for Improving Scientific Research Capabilities and Willingness of Initiative Scientific Research Innovation

Scientific research and innovation capabilities are the core of scientific research, such as people’s ability to explore unknown things, ability to discover new knowledge, and ability to apply new technologies (Tierney and Lanford, 2016). To measure the researchers’ capacity for scientific research, the internal quality and external scientific research results are mainly adopted. Internal quality mainly includes research ethics, methods and skills, scientific thinking, and research implementation ability (Zhang et al., 2011). The improvement of scientific research ability is affected by many factors, and the condition of these factors is the condition for improving the scientific research ability of graduate students.

When analyzing the influencing factors of graduate students’ scientific research and innovation, the advisor factors, such as academic status, academic experience, and allocation of energy show great association with doctoral graduate performance (McAlpine et al., 2020). At the same time, training and experience, opportunity and resources are proved to be the further pre-conditions for research performance (Bazeley, 2010). Tao Yuchun and others confirmed through quantitative research that factors, such as open academic environment, social support and guidance, tutor team, and innovation achievements have a significant impact on graduate students’ innovative ability (Tao et al., 2021).

In conclusion, high performance in research field need some circumstances, such as management and culture, as well as pre-conditions, such as knowledge foundation, these are the conditions for research work. Good conditions for improving scientific research ability are conducive in giving postgraduate students an excellent foundation for innovation ability, and will boost graduate students’ confidence in scientific research and innovation; otherwise, it will restrain the innovation abilities of graduate students. Based on this, the research hypothesis is proposed as follows:

Hypothesis 3. The ability of scientific research and innovation has a positive and significant impact on graduate students’ willingness to innovate in scientific research.

#### Hypothesis Based on the TBP

Corresponding academic interests to attitudes, regulatory pressures to subjective norms, and conditions for improving scientific research capabilities to perceptual behavior control, referring to the TPB theory, and the following research hypotheses are proposed:

Hypothesis 4: The behavioral intention of initiative scientific research and innovation has a positive and significant impact on initiative scientific research and innovation behavior.

Hypothesis 5: Academic interest has a positive and significant impact on initiative scientific research and innovation behavior through the behavioral intention of initiative scientific research and innovation.

Hypothesis 6: Regulatory pressure has a positive and significant impact on initiative scientific research and innovation behavior through the behavioral intention of initiative scientific research and innovation.

Hypothesis 7: Conditions for improving scientific research capabilities have a positive and significant impact on initiative scientific research and innovation behavior through the behavioral intention of initiative scientific research and innovation.

#### Moderating Effect

The interaction and communication between scholars and community are always considered as important variables leading to high performance in research work. A higher degree of interaction with other researchers (Kyvik and Smeby, 1994), professional networks (Brocato, 2001) and the network of communication with colleagues (Hedjazi and Behravan, 2011; Aydin, 2017) are paid attention to when research performance is studied. At the same time, to high-performance researcher, the meaning of being a researcher is the awareness of respect, concrete products (publication and citation), academic standing, and personal understanding and benefits to the community (Akerlind, 2008). The core issue of scientific research is not only to pursue the advancement of scientific research achievements, but also to pursue the transformation, promotion, and application of scientific research results. If scientific research is innovative, if it is not popularized, it will have no practical effect on the national economy and life. The transformation of scientific and technological achievements refers to the activities of subsequent testing, development, application, and popularization of scientific and technological achievements with practical value resulting from scientific research and technological development to the formation of new products, new processes, and new materials and the development of new industries for the purpose of improving the level of productive forces (Lite, 2019).

It is indicated that based on the selection of outstanding scientific research achievements, the government and administrative departments need to pay attention to the publicity and promotion of the achievements, and promote the higher level of scientific research through the establishment of results compilation and promotion, conference promotion, network platform promotion, and training promotion, etc. (Zeng and Yang, 2018).

Moderating variables are variables that affect the intensity and direction of the independent variables and dependent variables. The significance of the moderating variables is to identify the boundary conditions of the independent variables corresponding to the variables.

In summary, the promotion of achievements in scientific research will have an impact on the level of scientific research and the advancement of scientific payoffs, and scientific research and innovation behavior is a necessary component of the level and advancement of scientific research. The influence of the intention of initiative research innovation behavior on the initiative research innovation behavior will be interfered by the promotion of achievements in scientific research, which is the moderating variable of the model and plays a conditional role. Based on this, the research hypothesis is proposed as follows:

Hypothesis 8. The promotion of achievements in scientific research has a moderating effect on the relationship between behavioral intention of initiative scientific research and innovation and initiative scientific research and innovation behavior.

**FIGURE 1:**
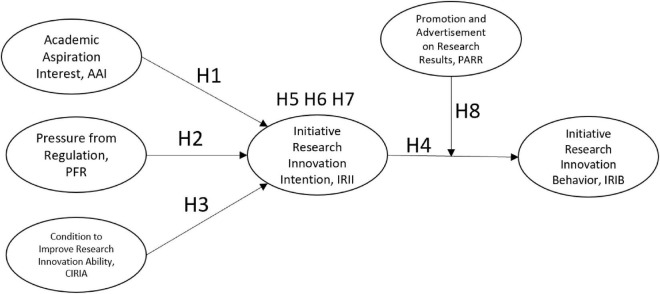
A theoretical framework of the research.

Based on the above assumptions, this study constructs the research model shown in Figure 1.

## Research Method and Material

### Sample and Procedure

This study focused on Chinese academic postgraduate students. A total of 684 students were selected. An online survey through the App of Wechat was conducted by inviting qualified postgraduate students to fill in and distribute the electronic questionnaire to the corresponding graduate class group. Rather than asking respondents simply whether they agree or accept an opinion statement, Likert scale items asked how strongly they agree or disagree with it, usually on a 7-point scale from 1 (= strongly disagree) to 7 (= strongly agree), with 4 being a neutral feeling or category. From September 10 to September 17, 2021, the research team distributed questionnaires through WeChat moments inviting qualified postgraduate students to fill in and distribute them to the corresponding graduate class group, excluding 83 questionnaires with incomplete fillings and inconsistent answer options, and finally recovering 684 valid questionnaires.

### Questionnaire Design

#### Composition of the Questionnaire

The research questionnaire is divided into two parts: the first part is the willingness and behavior of initiative research and innovation of postgraduate students and its influencing factors, which can be divided into seven dimensions, such as the willingness of initiative scientific research and innovation, initiative scientific research and innovation behavior, academic interest, regulatory pressure, scientific research and innovation capabilities, conditions for improving scientific research and innovation capabilities, and promotion of scientific research achievements. The questionnaire is designed using a Likert’s seven-level scale. In this scale, “1” means strongly disagree, and “7” means strongly agree. The second part is the basic personal information of the respondents, such as gender, grade, professional category, and school category. After the questionnaire design is completed, it was reviewed by industry experts and scholars, and was gradually revised to perfection.

#### Item Design of Latent Variable Measurement

##### Item Design of Variable Measurement Questions for the Willingness to Take Initiative in Scientific Research and Innovation

Mainly referring to the indicators in the research of Michael E. Burns et al. (2017) on the influence of positive evaluation factors of tutors on the positive interaction mode between students and teachers, combined with the research scene of initiative research willingness, referring to the index system of students’ willingness to initiatively interact with teachers, the index content of this variable is rewritten as follows: whether the scientific research and innovation work is included in the study plan, whether the scientific research and innovation work will be carried out seriously in the current period, and whether the scientific research work is also liked during holidays.

##### Item Design of Variable Measurement of Initiative Scientific Research and Innovation Behavior

In the research on the concept definition and scale development of scientific and technical personnel’s initiative innovation behavior, Zhao et al. (2014) designed 4 first-level indicators and 27 observable values related to scientific and technical personnel’s initiative innovation behavior, removing 5 values in the research that have been quantified and proved to be insignificant, combined with the research and innovation behavior of graduate students, this manuscript selected 12 indicators based on the research.

##### Academic Interest

In Bohan et al. (2019) designed 10 indicators corresponding to academic interest and life ambition in their research on the influencing factors of academic postgraduates’ academic interests, Lee and Durksen (2018) studied the dimensions of academic interest among undergraduate students. Referring to Lee’s indicator system, combined with Liu’s focus, this manuscript selected 8 indicators from 10 indicators in the academic interest measurement of postgraduates, reducing two indicators related to ambitions in life, constructed an index to measure the academic interest of postgraduates.

##### Regulatory Pressure

In Liu et al. (2021) studied the impact of the scientific research incentive system on graduate students’ innovative motivation taking self-efficacy as an intermediary factor. Four items were designed to measure the regulatory pressure on graduate students’ innovation. Aydin (2017) looked into the research performance of higher education institutions and developed the questions about the features of institutional structure. This manuscript directly used this indicator system for reference.

##### Conditions for Improving Scientific Research and Innovation Capabilities

In Tao et al. (2021) conducted a research on the influencing factors of the innovation capacities of postgraduate students in Jiangxi Province, and measured the conditions for improving scientific research ability from the aspects of open academic environment, social support and guidance, and tutor team. This manuscript drew on 11 measurement indexes in the research.

##### Promotion of Achievements in Scientific Research

In Zeng and Yang (2018) conducted research on the mission, basic characteristics, and promotion strategies of strong scientific research universities. They evaluated the promotion of achievements in scientific research from two aspects: the form of promotion of achievements in scientific research and the promotion and reward systems. This manuscript drew on the four measurement indicators designed by the research.

To sum up: The final questionnaire contains 6 dimensions, and 48 measurement indicators, which are shown in Table 1 below.

**TABLE 1:** Summary of dimensional measurement methods.

Variables	Index	Index content	Literature resources
Willingness to take initiative in scientific research and innovation	IIRI1	1. Have incorporated scientific research and innovation into my study plan	Burns et al., 2017
	IIRI2	2. I will seriously carry out scientific research and innovation this semester	
	IIRI3	3. I also like to do scientific research and innovation work during holidays	
	RAI2	2. I am more passionate about academics than other things	
	RAI3	3. Academics bring me great satisfaction	
	RAI4	4. When I introduced myself, I first thought of calling myself a “graduate who is doing academic research”	
	RAI5	5. I think working in academic society is a part of my life (whether as a career or as an interest)	
	RAI6	6. The idea of engaging in academic research is always in my mind	
	RAI7	7. Even if I don’t have academic work currently on hand, I still think about academic studies.	
	RAI8	8. Engaging in academics has brought me a strong sense of satisfaction and touch	
Initiative scientific research and innovation behavior	IRIB1	1. I can keenly discover problems that need improvement in scientific research	Zhao et al., 2014
	IRIB2	2. In order to solve the problems encountered in scientific research, I will take the initiative to make suggestions	
	IRIB3	3. I have a strong interest in scientific research and innovation, and I want to innovate from the bottom of my heart	
	IRIB4	4. In the process of scientific research and innovation, I am good at listening to other people’s suggestions	
	IRIB5	5. In scientific research, I welcome the collision of different ideas	
	IRIB6	6. Before scientific research and innovation, I will learn from other successful cases and find out way	
	IRIB7	7. I will anticipate the problems that may arise in scientific research and innovation, and find a solution	
	IRIB8	8. I am not afraid of failure in scientific research and innovation	
	IRIB9	9. When I encounter difficulties in the process of scientific research, I will try to solve them instead of avoiding them	
	IRIB10	10. I can be patient and repeat scientific research experiments and study processes over and over again	
	IRIB11	11. After repeated failures in scientific research, I will not give up easily	
	IRIB12	12. In the process of scientific research and innovation, I firmly believe that the goal will be achieved	
Research academic interest	RAI1	1. I am passionate about academics	Lee and Durksen, 2018
	RAI2	2. I am more passionate about academics than other things	
	RAI3	3. Academics bring me great satisfaction	
	RAI4	4. When I introduced myself, I first thought of calling myself a “graduate who is doing academic research”	
	RAI5	5. I think working in academic society is a part of my life (whether as a career or as an interest)	
	RAI6	6. The idea of engaging in academic research is always in my mind	
	RAI7	7. Even if I don’t have academic work currently on hand, I still think about academic studies.	
	RAI8	8. Engaging in academics has brought me a strong sense of satisfaction and touch	
Regulatory pressure	RP1	1. I feel pressure to qualify for graduation	Aydin, 2017; Liu et al., 2021
	RP2	2. I feel pressure to write my graduation thesis	
	RP3	3. Engaging in scientific research is a requirement of the college and school	
	RP4	4. Only scientific research results can get scientific research funding	
Conditions for improving scientific research and innovation capabilities	CIAR1	1. My college has a perfect graduation thesis evaluation system	Tao et al. (2021)
	CIAR2	2. The hardware and software facilities of our college can meet the training needs	
	CIAR3	3. My college has a democratic and free academic atmosphere	
	CIAR4	4. My school has frequent school-level academic exchanges and cooperation	
	CIAR5	5. My college and school encourage innovations	
	CIAR6	6. My college has a school-enterprise cooperation to provide an integrated platform for industry-university-research cooperation	
	CIAR7	7. I have little employment pressure	
	CIAR8	8. The instructor adopts heuristic teaching	
	CIAR9	9. Mentors provide many opportunities to participate in scientific research projects	
	CIAR10	10. Teachers value innovation ability in student evaluation	
	CIAR11	11. Mentors encourage novel ideas and creativity in scientific research	
Promotion of achievements in scientific research	PAR1	1. My school will hold lectures or hold press conferences to promote scientific research and academic achievements	Zeng and Yang (2018)
	PAR2	2. My college and school will compile the achievement promotion manual to promote the achievements	
	PAR3	3. My college and school have a reward system for the promotion of academic achievements	
	PAR4	4. There are professional scientific research achievement promotion agencies or platforms in my school and university	

### Data Analysis

The data analysis of this study is carried out in three stages: descriptive analysis, measurement model verification, and SEM. Descriptive statistics includes statistical analysis of population variables, as well as calculating the mean and SD of each variable to understand the degree of concentration of the variables; then, two-step analysis is used to carry out the measurement model and the structural model (Anderson and Gerbing, 1988), and confirmatory factor analysis (CFA) is performed to confirm the reliability and validity of the item, such as the composite reliability is used to measure the degree of internal consistency of various variables, convergent validity, and discriminant validity.

In the third stage, the SEM was used for analysis to test the goodness for fit of the research model, and then to verify the various hypotheses of the research framework. The SEM includes factor analysis, path analysis, and moderate effect analysis.

### Descriptive Statistics

The basic data of this research includes four items: gender, grade, major, and school level (as shown in Table 1). Among them, the gender is mainly female, with 426 students accounting for 62.4%; the grade is mainly the second year, with 253 students accounting for 33.51%; the major is mainly science and engineering, with 238 students accounting for 34.8%; the school level is mainly Project 211 (Project 985 and Project 211 refer to the higher education policy implemented by the Ministry of Education of the People’s Republic of China, 2020, and the selected universities all aim to develop into world-class universities), with 274 students accounting for 40.1% as Table 2 below shows.

**TABLE 2:** Analysis of demographic variables.

Characteristic	Category	Person/time	Percentage
Gender	Male	257	37.6
	Female	426	62.4
Grade	First Year	201	29.4
	Second Year	253	37.0
	Third Year	229	33.5
Subject	Literature, history, philosophy, and art	209	30.6
	Business (economic management)	149	21.8
	Agriculture and forestry medicine	87	12.7
	Science and engineering	238	34.8
University category	985	187	27.4
	211	274	40.1
	Private schools	205	30.0
	others	17	2.5

*N = 683. Project 985 and Project 211 refer to the higher education policy implemented by the Ministry of Education of China, and the selected universities all aim to develop into world-class universities.*

### Reliability and Validity Analysis

As shown in the table below, the standardized factor loading of the model is between 0.623 and 0.894, which is within the range, showing that each item has item reliability, acccording to Nunnally and Bernstein (1994) and Chin (1998); the construct reliability of the research variables is between 0.851 and 0.959, all exceeding 0.7. All meet the standards recommended by scholars, showing that each variable has good internal consistency; finally, the average variance extracted range is 0.578–0.746, higher than 0.5, meeting the standards proposed by Fornell and Larcker (1981) and Hair et al. (1998), showing that each variable has good convergent validity as Table 3 below shows.

**TABLE 3:** Analysis of measurement mode results.

Construct	Item	Significance of estimated parameters				Item reliability		Construct reliability	Convergence validity
					
		Unstd	S.E.	Unstd./S.E	*p*-value *p*	Std.	SMC	CR	AVE
Willingness of initiative scientific research innovation	IRI1	1.000				0.876	0.767	0.851	0.658
	IRI2	0.958	0.033	28.774	0.000	0.864	0.746		
	IRI3	0.923	0.048	19.039	0.000	0.678	0.460		
Initiative scientific research and innovation behavior	IRIB1	1.000				0.706	0.498	0.942	0.578
	IRIB2	1.039	0.053	19.727	0.000	0.768	0.590		
	IRIB3	1.176	0.060	19.696	0.000	0.768	0.590		
	IRIB4	0.820	0.046	17.651	0.000	0.700	0.490		
	IRIB5	0.863	0.048	18.037	0.000	0.718	0.516		
	IRIB6	0.823	0.047	17.661	0.000	0.701	0.491		
	IRIB7	1.029	0.054	19.166	0.000	0.756	0.572		
	IRIB8	1.114	0.065	17.072	0.000	0.680	0.462		
	IRIB9	1.046	0.052	20.256	0.000	0.813	0.661		
	IRIB10	1.165	0.055	21.019	0.000	0.842	0.709		
	IRIB11	1.190	0.057	20.802	0.000	0.833	0.694		
	IRIB12	1.133	0.056	20.355	0.000	0.812	0.659		
Academic interest	AAI1	1.000				0.861	0.741	0.959	0.746
	AAI2	1.074	0.036	29.908	0.000	0.849	0.721		
	AAI3	0.983	0.032	30.844	0.000	0.862	0.743		
	AAI4	1.084	0.041	26.563	0.000	0.800	0.640		
	AAI5	1.130	0.035	31.868	0.000	0.885	0.783		
	AAI6	1.150	0.036	32.145	0.000	0.890	0.792		
	AAI7	1.135	0.036	31.562	0.000	0.883	0.780		
	AAI8	1.075	0.034	31.414	0.000	0.876	0.767		
Regulatory pressure	PFR1	1.000				0.844	0.712	0.863	0.614
	PFR2	0.978	0.036	27.415	0.000	0.890	0.792		
	PFR3	0.800	0.039	20.525	0.000	0.727	0.529		
	PFR4	0.693	0.039	17.773	0.000	0.651	0.424		
Conditions for improving scientific research capabilities	CIRIA1	1.000				0.834	0.696	0.949	0.628
	CIRIA2	1.069	0.037	28.711	0.000	0.857	0.734		
	CIRIA3	1.081	0.035	30.620	0.000	0.894	0.799		
	CIRIA4	1.090	0.039	27.746	0.000	0.846	0.716		
	CIRIA5	0.958	0.034	28.086	0.000	0.851	0.724		
	CIRIA6	1.072	0.040	26.938	0.000	0.832	0.692		
	CIRIA7	0.813	0.045	17.935	0.000	0.623	0.388		
	CIRIA8	0.943	0.041	22.977	0.000	0.751	0.564		
	CIRIA9	0.922	0.042	21.890	0.000	0.726	0.527		
	CIRIA10	0.810	0.036	22.258	0.000	0.734	0.539		
	CIRIA11	0.827	0.038	21.990	0.000	0.728	0.530		

*Unstd., Unstandardized factor loadings; Std, Standardized factor loadings; SMC, Square Multiple Correlations; CR, Composite Reliability; AVE, Average Variance Extracted.*

Confidence interval (*CI*) is used to test the discriminant validity, according to Bootstrap, the two dimensions are discriminative valid when the *CI* is less than 1 (Torkzadeh et al., 2003). In this research, the bias-corrected and percentile method are used to test the *CI* and the result is shown as the following Table 4, all the *CI*s are less than 1, which shows that all dimensions are discriminative valid.

**TABLE 4:** Discriminant validity of the measurement model.

				Bias-corrected		Percentile method	
	Parameter		Estimate	Lower	Upper	Lower	Upper
IRI	<–>	IRIB	0.774	0.720	0.823	0.721	0.826
IRI	<–>	AAI	0.698	0.636	0.760	0.632	0.759
IRI	<–>	PFR	0.221	0.108	0.326	0.109	0.328
IRI	<–>	CIRIA	0.581	0.504	0.658	0.502	0.656
IRIB	<–>	AAI	0.785	0.734	0.834	0.731	0.831
IRIB	<–>	PFR	0.205	0.093	0.301	0.097	0.303
IRIB	<–>	CIRIA	0.627	0.560	0.693	0.559	0.692
AAI	<–>	PFR	0.174	0.073	0.274	0.074	0.275
AAI	<–>	CIRIA	0.583	0.513	0.644	0.511	0.643
PFR	<–>	CIRIA	0.222	0.109	0.327	0.116	0.333

*AAI, Active Academic interest; PFR, Pressure from regulation; CIRIA, conditions for improving scientific research capabilities; IRI, willingness to take initiative in scientific research and innovation; IRIB, Initiative research and innovation behavior.*

### Fit Analysis

In this study, the fit index refers to 194 SSCI papers discussed by Jackson et al. (2009), which serve as the blueprint for the analysis of model fit, and the most extensive nine fit indexes are used by this manuscript. Since the SEM sample is larger than 200, it is easy to cause the chi-square value to be too large and lead to poor fit, so the fit value needs to be corrected by the Bootstrap method (Bollen and Stine, 1992). The comparison table of the fit degree results of the Bollen-Stine Bootstrap modified model is shown in Table 5. Applying the Bollen-Stine Bootstrapping method, the goodness of fit index of this research shows that the results of this research are acceptable mode.

**TABLE 5:** Goodness of fit index.

Fit indices	Tolerance range	Fit measure	Fit discrimination
Chi-square		959.566	
Degree of freedom		658	
CFI	>0.9	0.987	Pass
RMSEA	<0.08	0.026	Pass
TLI	>0.9	0.986	Pass
GFI	>0.9	0.960	Pass
NFI	>0.9	0.902	Pass
χ^2^/*df*	<3	1.457	Pass
AGFI	>0.9	0.954	Pass

### Structural Equation Model Analysis

#### Path Analysis

**FIGURE 2:**
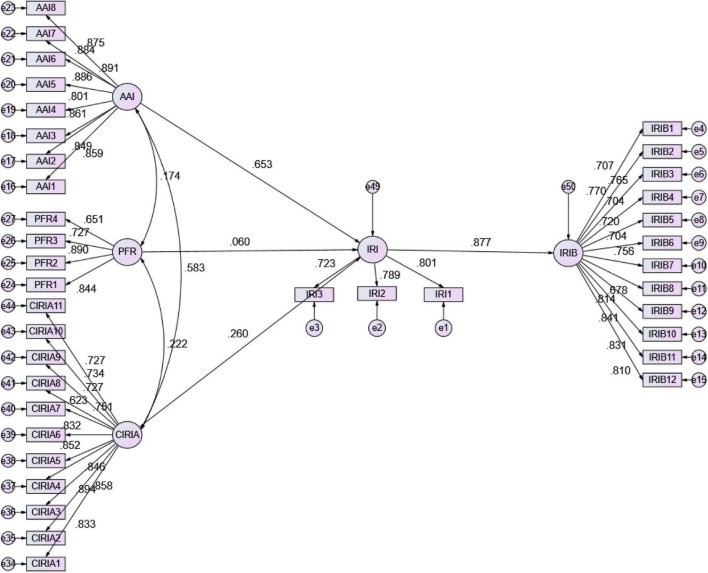
Statistical model diagram. AAI, Academic interest; PFR, regulatory pressure; CIRIA, conditions for improving scientific research capabilities; IRI, willingness to take initiative in scientific research and innovation; IRIB, initiative scientific research and innovation behavior.

The path coefficient results can be seen from the Figure 2 and Table 6 below. The research results support the research questions of this model. The interpretation effect of academic interest, regulatory pressure, and conditions for improving scientific research capabilities for the willingness to take initiative in scientific research and innovation is 71.7%. The interpretation effect of willingness to take initiative in scientific research and innovation for initiative scientific research and innovation behavior is 76.9%.

**TABLE 6:** Path analysis.

DV	IV	Unstd	S.E.	Unstd./S.E	*p*-value	Std.	R^2^
IRI	AAI	0.594	0.035	16.743	0.000	0.653	0.717
	PFR	0.048	0.022	2.148	0.032	0.060	
	CIRIA	0.261	0.035	7.431	0.000	0.260	
IRIB	IRI	0.749	0.045	16.678	0.000	0.877	0.769

*AAI, Active Academic interest; PFR, Pressure from regulation; CIRIA, conditions for improving scientific research capabilities; IRI, willingness to take initiative in scientific research and innovation; IRIB, Initiative research and innovation behavior.*

#### Mediation Effect and Moderation Effect Analysis

From the indirect effect analysis table of the mediation model in the following Table 5, in the indirect effect of academic interest → willingness to take initiative in scientific research and innovation → initiative scientific research and innovation behavior, *p* < 0.05, and this *CI* does not include 0 [0.357–0.535], indicating that the indirect effect is created and the intermediary effect is established. In the indirect effect of regulatory pressure → initiative scientific research and innovation behavior, *p* ≥ 0.05, and this *CI* includes 0 [−0.004 to 0.084], indicating that the indirect effect is not valid. In the total indirect effect of conditions for improving scientific research capabilities → willingness to take initiative in scientific research and innovation → initiative scientific research and innovation behavior, *p* < 0.05, and this *CI* does not include 0 [0.127–0.274], indicating that the indirect effect is created and the intermediary effect is established as the Table 7 below shows.

**TABLE 7:** Analysis of moderating effect.

DV	IV	Estimate	S.E.	*z*-value	*p*-value
IRIB	IRI	0.645	0.076	8.498	0.000
	PARR	0.128	0.043	2.980	0.003
	IRI	−0.033	0.015	−2.221	0.026

*AAI, Active Academic interest; PFR, Pressure from regulation; CIRIA, conditions for improving scientific research capabilities; IRI, willingness to take initiative in scientific research and innovation; IRIB, Initiative research and innovation behavior; PARR, Promotion and advertisement on research result.*

**FIGURE 3:**
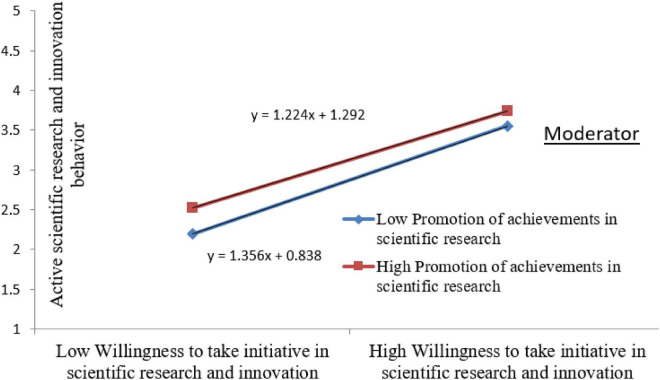
A sketch map of moderating effect.

In this research model, promotion of achievements in scientific research is taken as a moderator variable. The following table shows that the moderating effect of the promotion of achievements in scientific research on the willingness to take initiative in scientific research and innovation * active scientific research and innovation behavior is −0.033 (*z* = ∣−2.221∣ > 1.96, *p* = 0.026 < 0.05), which means that the moderating effect exists, and when the moderating variable (promotion of achievements in scientific research) increases by 1 unit, the slope of willingness to take initiative in scientific research and innovation to active research and innovation behavior will decrease by −0.033 units. As Figure 3 shows.

## Conclusion

### Discussion

This study mainly explores the initiative scientific research and innovation behavior of postgraduate students. Academic interest, regulatory pressure, and conditions for improving scientific research capabilities give postgraduate students the motivation, pressure, and ability to engage in scientific research and innovation. Among them, academic interest and conditions for improving scientific research capabilities have a very significant impact on the willingness to take initiative in scientific research and innovation which are consistent with the conclusion verified by Bland et al. (2006), Hedjazi and Behravan (2011), and Arastaman and Zdemir (2019) that scientific research interests have a positive and significant impact on the willingness of scientific research and innovation, and the conclusion of Ma et al. (2019) and Tao et al. (2020) that the improvement of scientific research capabilities can positively and significantly promote the formation of scientific research motivation. However, the significance of regulatory pressure is relatively low, and its non-standardized regression coefficient is only 0.048, less than 10% of the correlation coefficient of academic interests. Studies show that regulatory pressure does not have a significant direct effect on the motivation of scientific research (Liu et al., 2021). The reason may be that the regulatory pressure brought by the scientific research incentive system is too large, which weakens the confidence in scientific research. The conclusions of this study are different from this conclusion, which may be due to differences in the survey objects and questionnaire samples not only include postgraduate students, but also doctoral students, whose sample proportion of doctoral students accounted for 7.07%. Some doctoral students could not cope with relatively excessive pressure and had a mindset of avoidance or resistance. Therefore, regulatory pressure did not have a positive impact on the motivation of initiative research innovation; the object of this study focuses on postgraduate students. Relatively speaking, the regulatory pressure does not work beyond the capacity of most students, so it has a positive effect on the motivation of scientific research, but the effect is not significant. This conclusion also reflects the internal logic consistency with the conclusion of related research. It can be seen that external rewards and punishments, such as graduation qualification, thesis writing, and research funding, actually affect the willingness of postgraduate students to initiatively engage in research and innovation, but the influence is relatively limited. The inner academic interest and passionate exploring orientation have a significant impact on the willingness of initiative research and innovation of postgraduate students.

The willingness to take initiative in scientific research and innovation has a positive and significant impact on initiative scientific research and innovation behavior. It is highly consistent with the research conclusions proposed by Liu et al. (2020) that autonomous (initiative) behavior is better than participatory (passive) behavior to stimulate the initiative research and innovation behavior. The promotion of achievements in scientific research has played a moderating role in the model, but it is quite different from the research design: the promotion of achievements in scientific research has played a “negative” moderating role in the influence of the willingness to take initiative in scientific research and innovation on the behavior of initiative scientific research and innovation.

It is generally believed that the stronger the promotion of achievements in scientific research, the more it can inspire the sense of achievement and enthusiasm of graduate students, the more it can promote the combination of industry-university-research cooperation and application, and give full play to the social and economic values of academic research, thereby more effectively stimulating the initiative scientific research and innovation behavior. However, the data and model analysis show that the situation is just the opposite. This seems to be inconsistent with the theory of system innovation that the combination of industry-university-research can effectively promote innovation.

The average score of the questionnaire on the promotion of achievements in scientific research is 5.02, which indicates that the respondents’ evaluation of the level of the promotion of achievements in scientific research in their schools is above average; and the average scores of the four measurement indicators of this variable are all above 4 points. It can be seen that the schools where postgraduate students study should have academic meetings to promote research achievements, compiled academic achievement manuals, awarded for promotion, and built industry-university-research platforms. However, due to the difference in the way of work input and focus, the achievements of influential and authoritative experts are mostly promoted in the process of academic achievement promotion, while the achievements of postgraduate students do not become the subject and focus of promotion. At the same time, the degree of master’s research innovation and the level of integration of theory and practice are also relatively low. If it is promoted, the social response may not be as good as that of professors with higher qualifications, which may undermine the academic confidence of postgraduates. Therefore, this factor has a certain negative impact on the research and innovation behavior of postgraduates.

### Theoretical Implications

Existing research mostly focuses on the analysis of factors affecting the scientific research capabilities of postgraduates, but lack the analysis of their scientific research and innovation behaviors and mechanisms. Based on a TPB model, this study theoretically contributed an innovative research framework. This empirical research data from postgraduate students fully verified the effectiveness of the TBP.

### Managerial Implications

The empirical research shows that the mean value of the initiative scientific research and innovation behavior variables of postgraduate students is 5.20, and there is still a lot of room for improvement. With reference to the research conclusions of the model, the training institutions should not only give necessary regulatory pressure, but also pay more attention to the cultivation of academic interests and the improvement of the conditions for the improvement of scientific research ability, so as to give postgraduate students the motivation and ability to carry out initiative scientific research and innovation, effectively stimulate the intrinsic motivation of students and their willingness to take the initiative in scientific research and innovation.

The integration of “industry-university-research cooperation and application” and the promotion of achievements in scientific research play an initiative role in promoting scientific research and innovation and the social benefits of scientific research achievements. In the promotion of achievements in scientific research carried out by universities and other institutions, we should not only promote the effective transformation of scientific research achievements, but also pay attention to the stimulation and guidance of the training objects, such as postgraduate students. Through the effective design and organization of the promotion of achievements in scientific research, the negative influence of activities on the self-evaluation of postgraduate students can be eliminated, and the positive attitude and sense of gain of students in the promotion of scientific research achievements can be strengthened. We should guide postgraduate students to understand the social value and significance of scientific research achievements, so as to stimulate students’ scientific research and innovation behavior, and help the effective training of graduate students.

## Limitations and Future Research

In the sample of this study, the proportion of female students is relatively high, accounting for 62.37%; the proportion of “Project 211” college students (40.12%) is larger than that of “Project 985” colleges (27.38%); the proportion of male students who prefer academic career and 985 universities who pay more attention to academic postgraduate training is relatively small. It may not be enough to present the attitude and intention of samples with stronger academic ideals, which constitutes the limitations of this study and can be the direction of further research. This model test shows that the promotion of achievements in scientific research has played a negative regulatory role in the influence of the willingness to take initiative in the scientific research and innovation of postgraduate students on initiative scientific research behavior. This is quite different from our usual expectations and can also become a direction in the future research.

## Data Availability Statement

The original contributions presented in the study are included in the article/supplementary material, further inquiries can be directed to the corresponding authors.

## Author Contributions

HW and CY: conceptualization and validation. HW: formal analysis and methodology. YZ: investigation. HW and MM: writing–original draft and writing, reviewing, translating, and editing. All authors have read and agreed to the published version of the manuscript.

## Conflict of Interest

The authors declare that the research was conducted in the absence of any commercial or financial relationships that could be construed as a potential conflict of interest.

## Publisher’s Note

All claims expressed in this article are solely those of the authors and do not necessarily represent those of their affiliated organizations, or those of the publisher, the editors and the reviewers. Any product that may be evaluated in this article, or claim that may be made by its manufacturer, is not guaranteed or endorsed by the publisher.
